# Recent advances in testicular germ cell tumours

**DOI:** 10.12703/r/10-67

**Published:** 2021-08-31

**Authors:** Teresa Mele, Alison Reid, Robert Huddart

**Affiliations:** 1 The Royal Marsden NHS Foundation Trust, Sutton, UK; 2 The Institute of Cancer Research, London, UK

**Keywords:** testicular germ cell tumours, germ cell cancer, genetic hallmarks, biomarkers, novel treatments

## Abstract

Testicular germ cell tumours (TGCTs) are the most common solid tumours in young men and have an excellent overall cure rate and prognosis. In most patients, localised disease is cured by surgery alone, and a minority of patients receive short-course adjuvant chemotherapy to reduce the risk of further relapse. Also, in about 80% of patients, metastatic disease can be cured by systemic cisplatin-based chemotherapy. Unfortunately, for a proportion of patients, the disease exhibits platinum resistance and relapse occurs. Despite further lines of systemic treatment, cure can be difficult to achieve in these patients and ultimately about 20% of them will die from disease progression. Addressing the mechanisms underpinning platinum resistance is critical to improving the survival and chances of cure for these patients. This review describes the latest advances in TGCT research, focusing on the identification of novel biomarkers, genetic characteristics and exploring novel treatments.

## Introduction

Testicular germ cell tumours (TGCTs) are the most common cancer in young adult men^1,2^. These tumours represent a rare neoplasm on a population scale (about 1% of all oncological diagnoses in men) and their incidence has been increasing progressively over the last three decades^3,4^.

TGCTs arise mostly in the testis but can develop in extra-gonadal sites, albeit rarely (2–5%). Histologically, they are classified as seminoma, non-seminoma (yolk sac, embryonal carcinoma, choriocarcinoma and teratoma) or mixed^5^.

In the last 40 years, excellent rates of cure and overall survival (OS) have been achieved, including in the metastatic setting, thanks to multimodality treatment but principally owing to the introduction of cisplatin-based chemotherapy regimens^6^. Unfortunately, in up to 30% of metastatic TGCT patients, their disease is not cured by initial systemic therapy and salvage treatment is required^7^. Limited therapeutic options are available for patients with platinum-refractory disease, and prognosis is dismal and cure rates are low (<5%)^8^.

Staging and prognosis assessment are crucial in the disease management of TGCTs. Since its publication in 1997, the International Germ Cell Cancer Collaborative Group (IGCCCG) prognostic classification has provided a valuable tool for patients with TGCTs in defining risk stratification on the basis of histology, marker levels and metastatic sites (Table 1)^9^.

**Table 1.** International Germ Cell Collaborative Group Prognostic Classification (1997).

	Good	Intermediate	Poor
Seminoma	Any primary site No NPVM	Any primary site NPVM	Not applicable
Non- seminoma	All criteria: Gonadal/RP primary No NPVM hCG < 5000 IU/L AFP < 1000 ng/mL LDH < 1.5 ULN	All criteria: Gonadal/RP primary No NPVM 5000 ≤ hCG ≤ 50,000 IU/L 1000 ≤ AFP ≤ 10,000 ng/mL 1.5 ≤ LDH ≤ 10 ULN	Any criteria: Mediastinal primary NPVM hCG > 50,000 IU/L AFP > 10,000 ng/mL LDH > 10 ULN

AFP, α-fetoprotein; hCG, human chorionic gonadotropin; LDH, lactate dehydrogenase; NPVM, non-pulmonary visceral metastasis; RP, retroperitoneal; ULN, upper limit of normal.

Notably, the IGCCCG classification in advanced non-seminoma was recently redefined, and encouraging results have arisen from patients who received treatment in more recent years (1990–2013) compared with historical data based on treatments delivered prior to the 1990s. In the updated series, progression-free survival (PFS) improved for poor-risk patients (5-year PFS of 54% versus 41%) whereas OS improved in all IGCCCG risk groups (5-year OS of 96%, 89% and 67% respectively in good, intermediate and poor prognosis). A new prognostic model that includes older age and presence of lung metastases as additional negative factors has been proposed^10^. Also, a lactate dehydrogenase (LDH) cut-off of 2.5 times the upper limit of normal has been proposed to refine the classification of seminoma patients without non-pulmonary visceral metastases (formerly included in the good-prognosis group) as their outcome reflects the IGCCCG intermediate-risk group^11^.

The PFS and OS improvements highlight progress in the management of TGCTs over the last 30 years but also confirm the need for further research focused on patients with cisplatin-refractory disease or relapse and those with late relapses (occurring 2 or more years after the completion of treatment).

This review discusses recent developments in testicular cancer biology and clinical management, focusing on the following areas:

- tumour markers and biomarkers
- genetic predisposition and hallmarks
- platinum-resistant disease and novel treatments.

## Tumour markers and biomarkers

Tumour serum protein markers (α-fetoprotein, human chorionic gonadotrophin and LDH) are widely used at diagnosis, in monitoring treatment response and in follow-up^12^. Additionally, their levels contribute to the risk stratification of patients with metastatic non-seminoma according to the IGCCCG prognostic classification^9^. However, in a significant proportion of patients, disease will be marker-negative and therefore tumour marker levels will not reflect disease burden^13^.

There is an unmet need for biomarker development in the following areas of clinical practice:

- improving diagnostic performance at disease outset
- using biomarkers to aid in identifying which stage 1 patients will relapse and should be offered adjuvant chemotherapy, sparing those who do not require it
- improving early detection of relapse, particularly in those patients who are non-secretors of the traditional TGCT markers; this may allow a reduction in the radiation burden on these young patients and reduce imaging costs
- identifying patients with residual active disease in post-chemotherapy masses.

Recent data suggest a promising role for circulating microRNAs (miRNAs) in addressing such questions. miRNAs are non-protein coding RNAs that regulate the expression of protein-coding genes.

There is evidence of significant overexpression in the tissue of specific miRNA clusters (miR-371-373 and miR-302) in all TGCTs, regardless of histological subtype, patient age or site of primary presentation, but not in normal tissue^14^. The identification of stable serum miRNAs has led to a number of studies aimed at identifying miRNAs at time of diagnosis and in response to treatment^15,16^.

A prospective multicentre study of 616 patients with primary diagnosed TGCTs and 258 controls has shown extremely high sensitivity (90.1%) and specificity (94%) of miRNA-371a-3p (M371 test by quantitative polymerase chain reaction) in all TGCT subgroups except teratoma^17^. The promising role of microRNA 371a-3p has also been investigated in a cohort of 24 low-stage chemotherapy-naïve patients undergoing retroperitoneal lymph node dissection, and results have been excellent (area under the curve [AUC] on receiver operating characteristic [ROC] analysis 0.965, sensitivity 100% and specificity 92%). However, miRNA was not predictive of pure teratoma^18^.

miRNA levels have also been investigated as predictors of residual viable disease at retroperitoneal lymph node dissection after chemotherapy in 82 patients with TGCTs, and results were positive (AUC 0.874)^19^.

Interestingly, in a recent pilot trial in 111 patients with TGCTs where miR-371a-3p expression was retrospectively and blindly analysed on prospectively obtained samples and compared against clinical events, extremely high sensitivity (96%) and specificity (100%) were demonstrated, along with a positive predictive value of 100% and a negative predictive value of 98% in predicting active TGCTs^20^.

A recent comprehensive review addresses differences among studies conducted to date and miRNA performance^21^. Circulating miRNAs are being validated in prospective randomised clinical trials (Table 2).

**Table 2.** MicroRNA (miRNA) evaluation in current prospective clinical trials.

Study	Type of study	Intervention	Line	Patients	Serum miRNA	Timing	ClinicalTrials. gov Identifier
AGCT1531	Phase III	Active Surveillance	NA	Low-risk Stage I	• Correlation of miRNA levels and stage I relapse	• Pre orchidectomy • Every 1 month × 3 • Every 3 months for 1 year • Every 6 months for 1 year	NCT03067181
		Carboplatin versus cisplatin (+ etoposide, bleomycin)	I	Standard-risk Metastatic	• Marker decline on treatment • Identification prognostic miRNAs	Not available	
UKP3BEP	Phase III randomised	Accelerated versus standard BEP (bleomycin, etoposide and platinum) chemotherapy	I	Intermediate/ poor-risk Metastatic/ Mediastinal primary	• Marker decline on treatment • Identification prognostic miRNAs	• Day 1 • Day 22 • Day 43 • End of treatment • 1 year	NCT02582697
SWOG S1823	Prospective cohort study	Surveillance	NA	Stage I	• Correlation of miR-371a-3p levels and stage I relapse	• Pre-orchidectomy • Post-orchidectomy • 3 monthly (for 2 years)	NCT04435756

NA, not applicable.

Although further follow-up, validation and standardisation are needed, the excellent performance of miRNAs in terms of sensitivity, specificity and short half-life in these studies shows promise for positively impacting on patient management.

## Genetic predisposition and hallmarks

Compared with other solid tumours, TGCTs have a strong inherited genetic basis which accounts for almost half of the disease risk^22^. As far as development is concerned, TGCTs derive from reprogramming of cells in the early embryo and the germline, which dysregulates their developmental potency. A recent model identified seven types of TGCT, each harbouring unique epigenetic features^23^.

Genome-wide association studies have shed significant light on the factors leading to TGCT heritability, which, however, is not yet fully elucidated. First, heritability is not related to a single high-penetrance risk locus but instead is highly polygenic, and up to 49 risk loci have been identified so far^24,25^. Second, the role of several signalling pathways, such as *KIT-KITLG* signalling and *DAZL* and *PRDM14* (both of which are involved in germ cell differentiation), has been identified as crucial to disease development and genomic integrity^26^.

Overall, TGCTs are characterised by a number of genetic hallmarks, which can be related to tumour development and can have an impact on treatment response and resistance.

Chromosomal abnormalities, such as increased copy number of chromosome p12 (mostly as isochromosome p12), which is a well-established pathognomonic factor in TGCTs, are common^27,28^.

Also, unlike the majority of solid tumours, TGCTs, owing to their embryonic origin, are known to harbour a very low mutational burden and typically exhibit a lack of recurrent somatic non-synonymous mutations (mean of 0.5 mutations per megabase)^29^.

The Cancer Genome Atlas Research Network analysis on 137 primary GCTs showed somatic mutations in *KIT* (18%), *KRAS* (14%), and *NRAS* (4%), exclusively in seminomas^30^.

Other identified mutations by whole-exome sequencing in 42 TGCT cases include the tumour suppressor gene *CDC27* (11.9%). Copy number analysis showed amplification of the spermatocyte development gene *FSIP2* (15.3%). In two patients with cisplatin-resistant disease, a missense *XRCC2* mutation was also identified^29^.

## Platinum-resistant disease and novel treatments

Platinum-resistant disease remains a management challenge in TGCTs, and there is no consensus on the optimum salvage treatment to achieve disease remission. Several platinum-based standard-dose chemotherapy regimens—VeIP (vinblastine + ifosfamide + cisplatin), VIP (etoposide + ifosfamide + cisplatin), TIP (paclitaxel + ifosfamide + cisplatin) and EP (etoposide + cisplatin)—are currently used in this setting^31–33^, as is high-dose chemotherapy followed by autologous bone marrow transplant^34–38^. The complexity and frequency of this situation mean that guidelines recommend that such treatment should be undertaken in specialist centres. Key to progress in this area is multicentre and multinational collaboration. This collaboration has been facilitated by the development of international germ cell tumour groups such as the International Global Germ Cell Tumor Collaborative Group (G3) and the Malignant Germ Cell International Consortium (MaGiC).

A key example of this is the international randomised phase 3 study (TIGER, ClinicalTrials.gov Identifier: NCT02375204) that is currently comparing conventional dose salvage treatment (TIP) with high-dose chemotherapy (two cycles of paclitaxel and ifosfamide followed by three cycles of carboplatin and etoposide). The study is actively recruiting and the results are long-awaited to inform the salvage treatment of TGCTs.

At a molecular level, platinum sensitivity and resistance have not been fully elucidated. It has been shown that platinum sensitivity depends highly on cisplatin-induced DNA damage (due to insufficient or inefficient nucleotide excision repair and double-strand break repair), intact p53 signalling^39,40^ and an increased chemotherapy-induced apoptotic response due to mitochondrial priming^40^.

*p53* mutations and *MDM2* amplifications have been identified in platinum-resistant TGCTs, particularly in those with adverse clinical features, and have been related to poor outcomes independent of the IGCCCG risk class^41^.

Recent whole-exome sequencing on platinum-resistant TGCTs, compared with platinum-sensitive tumours, has shown several hallmarks of platinum-resistant disease, including increasing copy number and structural aberrations and an increased frequency of mutations affecting *KIT*, *p53* and *WNT*/*CTNNB1* signalling genes as well as loss of pluripotency genes and hypermethylation^42^. For example, in a 2016 study^40^, apoptosis and pluripotency regulators *NANOG* and *POU5F1* (also known as *OCT3/4*) expressed in TGCTs were not expressed in metastatic tumour deposits or mediastinal GCTs resistant to chemotherapy. However, the exact impact of these changes and the impact on mitochondrial priming are unknown.

Further research is needed to address the molecular basis of cisplatin resistance, and the exploration of novel treatments is a priority to improve outcomes in resistant disease.

A comprehensive description of targeted and novel treatments explored in TGCTs is beyond the scope of this review and has been addressed by others^39,43^. As summarised in Table 3, pathways explored include VEGF/PDGF-mediated angiogenesis, receptor tyrosine kinases (for example, c-KIT and MET), mechanistic target of rapamycin (mTOR) signalling, cyclin-dependent kinases, and poly (ADP-ribose) polymerase (PARP)-mediated DNA repair. Early-phase trials exploring the role of these novel agents in refractory TGCTs have shown disappointing results overall. While acknowledging that these studies often include heavily pre-treated patients, some of these studies were terminated early because of futility and because the observed overall response rates and outcomes were extremely poor. At the same time, it should be recognised that most of these studies have been in unselected patients.

**Table 3.** Clinical trials on novel treatments in testicular germ cell tumours.

Category	Drug	Phase	Patients enrolled (evaluable)	Relative risk	Progression- free survival	Overall survival	Patient selection	ClinicalTrials. gov Identifier	Reference	Status
TKI										
PDGFR/VEGFR	Sunitinib	II	10	CR/PR 0% SD 50%	-	-	Unselected	NCT00453310	46	Completed
		II	33 (32)	PR 9% SD 41%	mPFS 2.0 months	mOS 3.8 months	Unselected	NCT00371553	45	Completed
		II	5	-	12-week PFS: 20.0%	-	Unselected	NCT00912912	47	Terminated (slow accrual)
PDGFR/VEGFR/ FGFR/c-KIT	Pazopanib	II	43	PR 4.7% SD 44.2%	3-month PFS 12.8% mPFS 2.5 months	1-year OS 28.5% mOS 5.3 months	Unselected	NCT01743482	44	Completed
PDGFR/VEGFR/RAF/ c-KIT	Sorafenib	II	18	CR/PR 0% SD 3/18 (>1 year)	-	-	Unselected	NCT00772694	48	Completed
MET	Tivantinib	II	27 (25)	CR/PR 0% SD 20.0%	12-week PFS 21% mPFS 1 month	mOS 6 months	Unselected	NCT01055067	49	Completed
c-KIT, BCR-ABL, PDGFR	Imatinib	II	6	CR/PR 0%	-	-	Selected	-	50	Terminated
		II	7	CR/PR/SD 0%	-	-	Selected	-	51	Terminated
Anti CD30										
	Brentuximab	II	9	CR 11.1% PR 11.1% SD 22.2%	3-month PFS 22.2% mPFS 1.5 months	6-month OS 77.8% mOS 8.0 months	Selected	NCT01851200	52	Completed
		II	7	CR 14.3% PR 14.3% SD 42.8%	-	-	Selected	NCT01461538	53	Completed
		II	18	-	-	-	Selected	NCT02689219	-	Terminated
mTOR inh										
	Everolimus	II	15	CR/PR 0%	12-week PFS 40.0% mPFS 1.7 months	mOS 3.6 months	Unselected	NCT01466231	54	Terminated
	Everolimus	II	25 (22)	CR/PR 0%	12-week PFS 0% mPFS 7.4 weeks	mOS 8.3 weeks	Unselected	NCT01242631	55	Completed
	Sirolimus + erlotinib	II	4	-	-	-	Unselected	NCT01962896	56	Terminated (low accrual)
PARP inh										
	Olaparib	II	18	CR/PR 0% SD 27.8%	12-week PFS 27.8%	12- month OS 27.8%	Unselected	NCT02533765	57	Active
	Veliparib + carboplatin + gemcitabine	II	-	-	-	-	Unselected	NCT02860819	-	Active
CDK inh										
	Palbociclib	II	30 (29)	CR/PR 0%	24-week PFS: 28% mPFS 11 weeks	-	Selected	NCT01037790	58,59	Completed
	Ribociclib [teratoma]	II R	10 (8 ribociclib : 2 placebo)	CR/PR 0%	24-month PFS 71% ribociclib 0% placebo	-	Unselected	NCT02300987	60	Terminated (low accrual)
	Ribociclib [teratoma]	II	-	-	-	-	Selected	NCT02187783		Completed
Hypo-methylation										
DNMT	Guadecitabine + cisplatin	I	14	ORR 23%	mPFS 1.7 months	mOS 7.8 months	Unselected	NCT02429466	61	Completed
DNMT	Guadecitabine + cisplatin + gemcitabine	I^a^	2	-	-	-	Unselected		62	Completed
Immunotherapy										
PDL1	Avelumab	II	8	CR/PR 0%	12-week PFS: 0% mPFS 0.9 months	mOS: 2.7 months	Unselected	NCT03403777	63,64	Terminated
PDL1	Atezolizumab	II	-	-	-	-	Unselected	NCT02458638	-	Completed
PDL1 + CTLA 4	Durvalumab + tremelimumab	II	-	-	-	-	Unselected	NCT03158064	-	Recruiting
PDL1 + CTLA 4	Durvalumab +/− tremelimumab	II R	22	-	-	-	Unselected	NCT03081923	65	Recruiting Durvalumab monotherapy closed to accrual
PD1	Pembrolizumab	II	12	CR/PR 0% SD 16.7%	-	-	Unselected	NCT02499952	66	Terminated
PD1	Pembrolizumab	II	12	-		-	Unselected	NCT02721732	67	Recruiting
PD1 + CTLA 4	Nivolumab +/− ipilimumab	II		-	-	-	Unselected	NCT02834013	68	Recruiting
PD1 + CTLA 4	Nivolumab + ipilimumab x 4 -> nivolumab maintenance	II	5	CR/PR 0% SD 20.0%	-	-	Unselected	NCT03333616	69	Recruiting
PD1 + VEGFR/MET + CTLA 4	Nivolumab + cabozantinib +/− ipilimumab	I	5	CR/PR 0%	-	-	Unselected	NCT02496208	70	Recruiting
Other targets										
Claudin 6	ASP1650	II	-	-	-	-	Unselected	NCT03760081	71	Completed
B-RAF/MEK	Dabrafenib + trametinib	II	-	-	-	-	Selected	NCT02034110	72	Active
ALDH	Cisplatin + disulfiram	II	-	-	-	-	Unselected	NCT03950830		Recruiting

^a^SGI-110 with cisplatin and gemcitabine chemotherapy in patients with bladder cancer (EudraCT: 2015-004062-29). CR, complete response; mOS, median overall survival; mPFS, median progression-free survival; PFS, progression-free survival; PR, partial response; SD, stable disease.

A phase II single-arm study of pazopanib, an angiogenesis-targeted treatment, in 43 patients who had progressed after two or more platinum-based regimens yielded a 3-month PFS of 12.8% and a 1-year OS of 28.5%^44^. Likewise, despite promising pre-clinical activity, sunitinib showed poor clinical results in patients with cisplatin-refractory disease (partial responses ranged from 0 to 9%)^45,46^.

The role of *KIT* in genetic predisposition and the frequency of somatic mutations suggested this as an attractive target. Unfortunately, the reported responses with the tyrosine kinase inhibitors imatinib and tivantinib were disappointing^49–51^. This lack of activity may reflect the molecular features of TGCTs, as most identified *KIT* mutations are localised on exon 17 and associate with imatinib resistance^41^. Likewise, a phase II study of everolimus reported disappointing results^55^. A previous phase II study in the same setting was terminated because of futility; there were no responses in the first 15 patients who received treatment^54^. Similar results were reported in phase II studies addressing the cyclin-dependent kinases *CDK4–6*^58–60^. The hypothesis that cisplatin-sensitive tumours may also be sensitive to PARP inhibitors has led to testing of these inhibitors but with little evidence of activity to date^57^.

The lack of impact of targeted therapies on the disease has prompted exploration of alternative approaches.

Some activity has been seen with the antibody conjugate brentuximab vedotin targeting the CD30 antigen. In a small cohort of heavily pre-treated patients with CD30-expressing TGCTs, 3-month PFS and 6-month OS rates were 22.2% and 77.8% respectively^52^. In a different case series of seven patients who received brentuximab vedotin, two patients achieved an objective response^53^.

PDL1 expression in a significant proportion of TGCTs has led to the exploration of checkpoint inhibitors despite the low tumour mutational burden of these cancers. However, studies of the PD1 inhibitor pembrolizumab and PDL1 inhibitors avelumab and durvalumab have failed to show clinical activity, and no responses were observed in TGCTs^63–67^.

Current studies are investigating PD1-directed agents alone or in combination (such as nivolumab + ipilimumab and durvalumab/tremelimumab)^68,69^. Cabozantinib +/− ipilimumab in a phase I study, however, showed no observed responses in the TGCT subgroup^70^.

Hypomethylating agents such as guadecitabine (SGI-110) have been suggested as potentially promising novel targets in view of the observed DNA hypermethylation exhibited by platinum-refractory TGCTs as opposed to platinum-sensitive tumours. In pre-clinical studies, TGCTs were extremely sensitive to low-dose decitabine, a DNA methyltransferase inhibitor, which restored sensitivity to cisplatin in cell lines^73^. A phase I study of guadecitabine and cisplatin in 14 patients with TGCTs showed an overall response rate of 23%^61^; excellent responses were also reported in two patients with platinum-refractory disease treated in a phase 1 study of cisplatin, gemcitabine and guadecitabine^62^.

It is still unknown to what extent the lack of activity observed with the majority of these agents is due to patient selection or to TGCT intrinsic biology. Certainly, further research on biomarkers is needed to identify novel treatments in refractory disease.

## Conclusions

TGCTs are a heterogeneous group of diseases which in general have excellent cure rates that have improved over the last three decades. Treatment and cure of cisplatin-refractory disease are challenging, and the optimal treatment for these patients is not yet clear. Further research on cisplatin resistance is needed to expand the current therapeutic options and to achieve better outcomes for patients with refractory disease.

Promising novel biomarkers are being investigated and should their role be fully validated, the management of patients with TGCTs will certainly evolve.

Given the complexity of its management and multimodality treatment, referral to high-volume centres is crucial. As with any rare disease, international scientific collaboration such as the International Global Germ Cell Tumor Collaborative Group (G3) and the Malignant Germ Cell International Consortium (MaGIC) is the key to allow faster advances in research and clinical practice. 
